# The sensemaking narratives of scientists working in health professions education scholarship units: The Canadian experience

**DOI:** 10.1007/s40037-020-00577-1

**Published:** 2020-05-11

**Authors:** Brittany Etmanski, Stanley J. Hamstra, Lara Varpio

**Affiliations:** 1 Department of Sociology & Legal Studies, University of Waterloo, Waterloo, ON Canada; 2 Accreditation Council for Graduate Medical Education, Chicago, IL USA; 3 Department of Medicine, Uniformed Services University of the Health Sciences, Bethesda, MD USA

**Keywords:** Career success, Research scientist, Health Professions Education Scholarship Units (HPESU)

## Abstract

**Introduction:**

To date, research studying health professions education scholarship units has overlooked the perspectives of research scientists in the field, despite their important role in these units. This research explores how health professions education scientists uphold and/or upend the institutional logics of the units they work within.

**Methods:**

Recruited via snowball sampling, 29 Canadian health professions education scientists participated in semi-structured interviews that lasted between 32–55 min. Data analysis was informed by the theories of organizational institutionalism—specifically, the microfoundation element of sensemaking.

**Results:**

Respondents’ narrations of career success were overtly linked to their research-oriented pursuits above other expectations (i.e., teaching, service).

**Discussion:**

Respondents’ narrative revealed a mismatch between the value they associated with teaching- and service-related pursuits, and the value the institution associated with those pursuits. Participants indicated a need to reconceptualize the institutional value associated with these endeavors.

**Electronic supplementary material:**

The online version of this article (10.1007/s40037-020-00577-1) contains supplementary material, which is available to authorized users.

## Introduction

A growing body of research is investigating health professions education scholarship units (HPESUs) [1–5]. HPESUs are defined as formally recognized academic units housed within medical, nursing, and other health professions schools, or within hospitals [1]. While the organizational structure of these units varies (e.g., unit, department, office), they share the mandate to support members of the housing institution (e.g., clinician educators, administrative leaders, research scientists) to engage in health professions education scholarship [1, 2]. An HPESU has been recognized as an essential requirement for institutions that educate health professionals since these units help the housing institution (a) educate higher numbers of clinicians (b) who can meet the demands of modern healthcare delivery, (c) while simultaneously meeting increased social accountability expectations [6]. Research has laid the foundation for on-going studies of these units; however, to date, research has been informed solely by the perspectives of unit directors and other leaders in health professions education [1–5]. This limited scope is problematic because health professions education scholarship scientists have been identified as vital contributors to the success of individual HPESUs [3]. A health professions education scholarship research scientist is defined as an individual working in health professions education, who holds a graduate level degree(s), and engages in scholarship related to health professions education [2]. This study aims to bring the perspectives of these scientists to the fore so that the success of HPESUs can be buoyed by insights from these important contributors. Indeed, without attending to the experiences and perceptions of these scientists, we risk neglecting a key piece of the engine that propels HPESUs and, through those units, the field of health professions education.

Given the research expectations that ground the field of health professions education [7], and the fact that research in this area requires investigatory skills that differ from those traditionally taught to healthcare providers [8], it was predictable that HPESUs would employ research scientists willing to emigrate from other fields (e.g., education, psychology, and rhetoric) [9]. The scientists working in these units engage in a range of scholarly activities, often in collaboration with clinician educators. Such partnerships connect research with practice in hopes of achieving the knowledge translation goals of applied research [10]. These partnerships have proven to be highly generative. A recent study of productivity in medical education research reveals that PhD-trained scientists are principal contributors to health professions education’s body of knowledge [11].

In addition to building the field’s evidence base, health professions education scholarship scientists shape the units in which they work through their decisions, values, and activities. These scientists actively mold the institutional logics that are entwined with and upheld in HPESUs [12]. Institutional logics, a concept developed by scholars in organizational science, are the socially constructed and historically developed pattern of beliefs, rules, and practices that act as organizing principles of an institution [12]. When applied to HPESUs, institutional logics are the assumptions and values that underpin the unit, providing a framework that shapes reasoning, offers criteria for legitimacy, and organizes the foundational expectations of those working within and with the unit[13]. Research has identified three logics informing HPESUs: the logic of financial accountability; the logic of a cohesive educational continuum; and the logic of academic research, service and teaching [1]. Scientists can maintain and reinforce these logics by aligning their work with the unit’s enactment of these logics; alternatively, scientists can weaken these logics by electing to work outside the unit’s areas of concentration. In short, health professions education scholarship scientists can support, amend, and/or subvert these logics that ground the HPESU itself, thereby exerting considerable influence on these units.

The purpose of this investigation was to explore how health professions education scholarship scientists uphold and/or upend the institutional logics that support HPESUs. Previous research identified the institutional logics enabling HPESUs by studying the definitions of success upheld by the directors of those units [1, 3]; we aligned our investigation to those studies. We asked: How do PhD-trained health professions education scholarship research scientists describe their success as faculty members in an HPESU? Using sensemaking—a microfoundation of institutional logics—as a theoretical lens to inform analyses, we examine these data to explore why these researchers narrate their careers in the ways that they do.

## Sensemaking: a microfoundation of institutional logics

Scholars investigating the microfoundations of institutional logics study how individuals locate themselves in logics, construct them, maintain them, transform them, and/or eschew them [14, 15]. One element of these microfoundations is called sensemaking—i.e., the process through which individuals interpret situations, generate understandings of situations, and craft justifications for future actions [16]. Individuals engage in and achieve sensemaking through narratives. These narratives manifest specific institutional logics and give evidence of how the individual is accepting, modifying, or rejecting them. In other words, institutional logics—and by extension, the HPESUs themselves—are activated in, constructed by, and transformed through sensemaking narratives [17].

In this study, we examine health professions education scholarship scientists’ narratives to explore the sensemaking activities that are realized therein. We focus on how specific institutional logics are promoted, transformed, and/or denied in these narratives.

## Methods

The research ethics board at the Ottawa Hospital approved this study (file number: 20180277-01H). We confirm that all procedures performed in studies involving human participants were in accordance with the ethical standards of the institutional and/or national research committee and with the 1964 Helsinki Declaration and its later amendments or comparable ethical standards. There were no potential harms to participants. All participants provided informed consent and anonymity was guaranteed.

### Design

This study is part of an international program of research investigating HPESUs rooted in a constructivist paradigm [1–5, 14, 18]. Data collection was conducted in the fall and winter of 2013. In 2014, LV and a research assistant analyzed the data using Bourdieu’s theories to inform analyses. For over a year, LV and the research assistant read and re-read the data, studied Bourdieu’s theories, and endeavored to develop new insights that would make a novel contribution to the field. These efforts were unsuccessful. Given that other branches of the program of research into HPESUs were progressing, the data from the Canadian research scientists was put aside in 2015, but they were not forgotten. During the summer of 2018, BE and LV decided to reconsider the data from the Canadian health professions education scholarship scientists using theories from organizational sciences—and specifically institutional logics and sensemaking narratives—to inform analysis.

### Participants

We sought to interview all the health professions education scholarship research scientists working in Canada. Given that no registry exists, we used snowball sampling to find participants [19, 20]. We began by interviewing scientists who were known to our research team. At the end of each interview, we asked participants to name other Canadian health professions education scholarship research scientists. We approached these nominees, asking them to participate in the study. If they agreed, we repeated the snowball technique at the end of these interviews. We repeated this process until no new names were nominated. Since the study was conducted in a constructivist orientation, the perspectives of the health professions education research scientists at our own institution (i.e., LV and SH) informed the data collection and analysis because they were investigators in the study. We decided not to interview these individuals as part of the data set since their insights were already informing the research. We invited all 32 nominees to participate in the study: 30 consented. One interview was omitted from the data set because the participant did not meet the criteria of being a health professions education research scientist. Therefore, 29 individuals participated in this study. All participants worked in HPESUs and represented all Canadian units that were standing at the time. In terms of the number of years working as health professions education research scientists, 15 had been engaged in the field for <5 years (51.7%), 5 for 5–10 years (17.2%), and 8 for >10 years (27.5%). Seventeen participants worked in units housing ≥3 scientists; 12 worked in units with ≤2 scientists.

### Data collection

We developed a semi-structured interview protocol, drawing on the protocol used to interview directors of Canadian HPESU directors about their definitions of success for their units (see Appendix online) [3]. A qualitatively trained research assistant conducted telephone interviews from August–October 2013. The interviews, which ranged from 32 to 55 min in duration, were audio recorded, and transcribed by an external, professional service. All identifying features were removed during transcription. The study’s principle investigator (LV) reviewed each transcript against the audio file for accuracy.

### Data analysis

Drawing on Saldaña’s approach, BE and LV employed first- and second-cycle coding methods. First, provisional and value coding were employed. Provisional coding was informed by previous research findings analyzing the same interview data (i.e., the initial 2014 coding), the overarching objectives of the current research, and authors’ experiences and areas of expertise [21]. While some of the original 2014 coding was interesting and relevant, the heart of this analysis emerged from the process of value coding. This coding process explores interpersonal and intrapersonal participant experiences by seeking to identify respondents’ world view through their narrated attitudes, values, and beliefs [21]. Through value coding, the codes capturing participants’ descriptions of tensions and affordances associated with career success were inductively identified as particularly important.

To explore the data in greater detail, BE and LV employed second-cycle theoretical coding. This method of coding identifies a core theoretical concept that represents the heart of the research, and that houses all other codes identified within the analysis [21]. This method of coding is applied to explore how a phenomenon develops, how it compares with others, and/or why it happens [21]. Our core theoretical framework focused on organizational institutionalism and institutional logics to explore what institutional and individual perceptions and assumption of success entailed. Coding nodes housed by this core theme were: mimetic isomorphism, institutionalization, and loose coupling. An institutional framework was chosen to highlight the organizational pressures and institutional expectations placed on health professions education research scientists. While the concepts highlighted some aspects of the data, BE and LV agreed that they failed to capture the core arguments and insights offered in the data.

In hopes of identifying a way to articulate the richness of the findings, LV reviewed the analyses, studied the theories and concepts of organizational institutionalism, and revised the core of the theoretical coding employed. An institutional framework was still employed, but the core of the coding approached shifted to focus on the microfoundations of institutional logics, specifically sensemaking. BE examined LV’s new revisions, verified the analysis, and concurred with the interpretations. In the last phases of analysis, BE and LV recursively verified analyses, confirmed data interpretations, and developed this manuscript. SH reviewed the analyses, confirmed the interpretations, and refined this manuscript.

## Results

Participant narratives accentuated the logic of academic research, service, and teaching. They narrated career development and success in terms of meeting requirements to engage in research. They also expressed the need to modify conceptualizations of service, and to transform perceptions of teaching. Participants described a strategy for meeting these competing elements of career of success while simultaneously satisfying the institution’s expectations for promotion.

### Supporting institutional success via research

All participants, those working in both large and small HPESUs, emphasized their career success in terms of a research-intensive focus. Participants explained that research success was heavily socialized in the traditions and culture of academia. The importance of research-intensive pursuits was a narrative that cut across decades of PhD-trained health professions education research scientists (see Tab. 1 for illustrative data excerpts).

**Table 1:** Data excerpts illustrating results

Supporting institutional success via research	“I think that there’s a fundamental academic currency of grantsmanship, publication, presentation, that defines, for me, success. And those are some of the traditional markers that any faculty member, regardless of where they would be situated would be looking at.” (P21: >10 yrs) “To be successful you have to pull in grants, you have to publish papers. If you’re junior you should be publishing first author papers; if you’re senior you should have a stable full of people that you are publishing senior author papers with. [pause] It’s sort of the traditional markers of academic success.” (P13: 6–10 yrs) “The typical model for measuring success has been amount of grant dollars you get and then the actual value of those grants; your number of publications, the impact factor of the journals for which you’re publishing, the quantity of your publications, particularly those in which you’re indicated as the senior author or principal author.” (P9: ≤5 years)
Modifying conceptualizations of service	“I think we are absolutely essential to the future of medical school. We are committed to education excellence. The only way that we achieve that is though educational scholarship and therefore, although we may be small, we are absolutely critical to the future success and well-being of all our educational programs and we really should be given more profile and more support in profiling that role. It’s not just we are yet another branch of scholarship. We play a rather different and more central role to the functioning of the organization …. It [the scholarly services offered by the HPESU] needs some [pause] legitimacy. It needs some funding. It needs to be positioned as a critical and essential part of how the medical school goes about doing what it’s doing and it needs to be connected therefore to the mission but also the quality assurance, the quality improvement of its programs.” (P17: >10 yrs) “We all do a lot of work behind the scenes, whether it’s contributing to how things work in the faculty or committees, or whatever, I don’t think any of that counts for any kind of academic success for anybody …. It’s all on our CVs but no one cares. So those are things that we do that are part of functioning [pause] If we don’t do them, you know, we lose the support of our academic institutions, but at the same time it’s like housework. You gotta do it but it doesn’t count.” (P13:6–10 yrs) “I don’t think any of it detracts. I think we have to remember that education informs research, which informs education, which [pause] I mean it’s all linked. I think education research and practice need to be linked and informed by one another and I think if I’m called on to do the odd faculty development session or to teach a course in a master’s program, I think that’s perfectly fine.” (P14: ≤5 years)
Transforming perceptions of teaching	“I think that for me, it all comes down to being able to be a valued contributor to the fundamental success that we set out in training physicians and other health professionals in [name of local institution]. And so that’s a big kind of generic statement, but you know, if I look at a specific example in curriculum renewal, being able to design and develop this new curriculum, I see a personal measure of my success as, you know, being a really strong and productive contributor to that mission, to really improve the curriculum.” (P21: >10 yrs) “I think that that’s [research productivity is] really important but, you know, I think from my standpoint, the actual applied part is important. Putting the theory into practice and having quality programs that are informed by research and by evaluation data.” (P26:6–10 yrs) “I’m increasing capacity for the department to be more scholarly, and that’s cutting across from the individual level all the way to the programmatic and departmental level …. So that teaching is much more anchored in evidence based, that it informs other kind of teaching that’s building blocked towards programming, so scholarly teaching. Capacity to research more effectively the questions and concerns that educators want to address in their own context. So increase skills in faculty and residents.” (P2: ≤5 years)

When articulating the essential status accorded to research-related activities to these scientists’ careers, respondents stressed the need to disseminate peer-reviewed publications. This participant’s comment exemplifies the emphasis placed on publication:

*I would say that number one, probably—well, I guess number one, being published in medical education, you know, prominent medical education journals in Canada and elsewhere. (P16)*

Peer-reviewed, evidence-based research manuscripts were described as tangible products of their research efforts that demonstrated the ability to be an effective faculty member. As one respondent’s off-hand comment illustrates, a successful career is equivalent to a recognized program of research—an outcome that is “*obviously*” (P25) secured through publications:

*I think success for a PhD is developing a program of research, meaning that other people at my institution and around the country, around the world, kind of know what it is that I study so they know my research area. They know my work. It would have to be obviously based on receiving a large number of publications. (P25)*

Respondents explained that they worked in HPESUs to engage in research. As one participant explained, career success for scientists working in these units was being allowed to run investigations with *“reckless engagement and passion” (P17)*. Research was the core element to participants’ conceptualizations of success.

### Modifying conceptualizations of service

While participants emphasized engagement in research as essential to career success, respondents cautioned that exclusively focusing on traditional metrics of research success was too narrow an application of the concepts of *research* and *scholarship*. Participant narratives highlighted that their personal definitions of career success encompassed activities that advanced the field of health professions education itself. As this participant explains, a broader definition, one that acknowledged how individuals developed and bolstered a “*culture of scholarship*” (P7), was emblematic of success:

*One of the things that I see myself as pleased to be able to point to is the extent to which I have participated in the larger endeavor of trying to raise the standard of scholarship that has gone on in the context of the larger field. That doesn’t necessarily manifest itself as individual papers or individual grants that I’ve received, rather just the general increase in the quality of the work that’s being done … I don’t think that we have good measures of those kinds of things, and that certainly doesn’t show up on my CV anywhere, but that is the stuff that gets me very excited. (P7)*

Developing a culture of scholarship was realized through engagement in scholarly activities outside of traditional research efforts, often specifically framing service-related pursuits as scholarly endeavors. Participants at every career stage echoed the importance of service work, lamenting that the larger institution did not value their service efforts. Respondents explained that their service offerings were vital to the success of their local institutions and were unique contributions that they offered to the institution (see Tab. 1 for illustrative data excerpts).

If research work was styled as the glamorous side of these scientists’ academic careers, service work was fashioned as the necessary *“housework” (P13).* Engaging in program evaluations, leading faculty development sessions, supporting learner assessments, sitting on committees, creating materials in support of accreditation: while these are not prestigious pursuits, the participants recognized their importance and were committed to engaging in this work. However, respondents warned that engaging in too much service work would be detrimental to their careers. As this participant explained, service activities could take too much time away from research:

*I spend an awful lot of time doing administrative types of things. Committee work and that kind of thing … It really is difficult to think and write about medical education and to theorize about medical education when you’re doing it between meetings. You know, it’s [research is] not the kind of thing that you can pop in and out of, at least for me. I need concentrated time in order to sit, to think and write and to conduct my research and synthesize that information, that kind of thing. Like I say, it’s hard to do that when you have a half an hour between committees. (P18)*

Protecting early-career scientists from becoming too involved in service activities was noted as an important function of the unit’s leadership, lest those junior faculty members not be able to meet the research expectations required for academic promotion:

*My time is protected. There are a lot of committees and a lot of administrative work, or administrative responsibilities out there in the department, and I know in the past people like myself have been asked to engage in a lot of that work. But thus far I’ve been protected by my department to really give me the time to concentrate on grant writing and writing up papers and getting the work done by which I will be ultimately evaluated. (P9)*

Respondent narrations highlighted the tension between service and research. Service work was described as time consuming, diverting time away from the research responsibilities that participants enjoyed and that were accorded greater institutional value. However, participants also narrated service work as a necessary component of keeping the institution running, and so should also be accorded institutional value. When asked to describe what obstructed their overall career success across all these commitments, this respondent’s ambiguous and self-contradicting observation articulated the service-research tension expressed by many participants:

*The service load I carry, the administrative service load I carry ….meetings that aren’t so focused on medical education that may be focused more on finance, student bursaries, student scholarships, all very important but they certainly make it difficult to do the—to have the time for education scholarship. Having time that’s not eroded by so many other things, so many administrative things. I think that’s right. On the other hand, one’s research is very enriched by the work around [the institution] and what else is going on, right? I don’t think [pause] you know [pause] medical education scholarship does not operate in a vacuum and you get your ideas and stimuli from the people with whom you work in whatever contexts. (P15)*

Participants struggled to strike a balance where they avoided engagement in so much service work that their research faltered, while also engaging in enough service work to make their contributions relevant to the institution. When narrating that goal, respondents articulated their programs of research as *applied* research and so relevant—even integral—to their service commitments:

*For us, it’s also an applied sense, because I’m not just doing puritan research for the sake of research. There is a lot of connection to program delivery and program evaluation. (P26)*

As one participant explained, the ideal situation was to harness service work to meet research goals:

*I think what’s most important is that there is a supportive environment within the program that allows me to conduct my research as well as integrating my research with the program needs so that I’m able to basically meet a couple of goals, all of them, all in the same stroke. (P31)*

### Transforming perceptions of teaching

Respondents also described the importance of teaching to their career success, even if the institution did not accord similar value to those activities. As one participant observed, despite the fact that HPESUs are located in institutions dedicated to the education of clinicians, most institutions did not value teaching:

*I also value the being a bit of an advocate for the importance of understanding education. One of the things, when we were talking about what the goals and missions were for the medical program, is to really get people to understand that* we are in the business of education, not in the business of medicine *[participant’s emphasis]. And that’s probably not always a well-accepted idea, but part of that is really to change the ethos to the fact that the Faculty of Medicine educates, and then those that they educate go out and do the business of medicine. (P21)*

Participants narrated valuing many teaching-related endeavors including: mentoring residents and clinician educators to engage in health professions education research; informing curricula with findings from educational research and theory; and supporting the institution’s teaching faculty through faculty development. Participants’ teaching work was not taking place in traditional health professions education space—e.g., not often in a classroom setting or at the bedside; instead, their teaching contributions were contributing to the institution’s culture of scholarship. For participants, this teaching work was essential to the institution’s success (see Tab. 1 for illustrative data excerpts).

Participants described engaging in teaching-the-teacher (i.e., supporting clinician educators who teach medical learners). Working with clinician educators to become more effective teachers or successful health professions education researchers generated powerful collaborations. However, respondents often avoided labeling their collaborators as *learners*. This was particularly evident in the comments from early-career scientists. They tended to describe being partners in these relationships, placing *“emphasis on collaboration” (P9) *and not on the mentoring work*:*

*When I say teaching, faculty development, etc., it means working with colleagues to help their research program, which also could include me as a collaborator. (P2)*

But this was a risky way of describing teaching efforts. Participants recognized that framing teaching activities as collaborations could threaten their academic promotion:

*Yeah, it’s [teaching is] not valued [laughs]. I mean I’ve actually been asked whether I wanted to be a faculty service officer instead of a tenure track faculty member on several occasions. [pause] The work that I do collaboratively with the clinical educators is not always publishable. So even though I’m guiding them or doing peer mentorship, they’re seeing that as a service. (P19)*

### Strategizing to realize success

Participants were aware that embracing research *and* service *and* teaching as important elements of career success required a strategic approach. As this participant explained, the scientists’ careers involved managing competing priorities:

*In the field that I’ve chosen [health professions education] we are pulled in multiple directions and have our own research programs as well as doing some consultation and supervision and serving on various administrative committees. So it’s mostly a matter of figuring out how to manage one’s own time and how to prioritize and ensure that those priorities are maintained. (P1)*

Participants described having developed strategies to meet these multiple priorities. The most common was to frame service and teaching activities as contributing to research aims. For example, respondents described: turning the service of helping to deliver a specific educational program into program evaluation research, and leveraging the teaching of clinician educators to be rigorous methodologists into a research collaboration.

But aligning service and teaching responsibilities with research required a key foundational premise to be in place: academic freedom. The scientists agreed that a core contributor to their career success was being empowered to make their own choices in their academic careers:

*Academic freedom, it’s the liberty, the freedom to do, to engage in any kind of question I want to engage in. (P3)*

Academic freedom required the trust and support of the leadership of the HPESU and the institution.

## Discussion

Health professions education scholarship research scientists’ descriptions of career success reflected how aspects of an institutional logic underpinning HPESUs—the logic of research, service and teaching—were supported and challenged. Respondents supported the logic of research; they positioned themselves as researchers. Their narratives about reconceptualising service activities and transforming perceptions of teaching reveal how their sensemaking aligned these activities to their research focus and supported the development of a culture of scholarship. By justifying service and teaching work as springboards for research, participants framed these activities as interconnected elements that could help propel their research. The leadership of the unit and/or the institution could facilitate those interconnections when they protected the researchers’ academic freedom.

Research suggests that sensemaking is the product and producer of power relations [16]. Some scholars suggest that power is inherent in sensemaking, asserting that “the creation of new understandings is not free of power issues and self-interested behaviour [22, p. 1629].” In light of this assertion, it is reasonable to interpret participants’ sensemaking as a means for leveraging the power available to them. The institutions in which HPESUs are housed are focused on graduating clinicians, and so they often have a mission that is focused on the end result of clinical readiness, not on the educational processes that support the achievement of that end. In these contexts, value is not accorded to service work and teaching; instead, research is deemed valuable. Given this situation, by aligning service and teaching activities with research goals, the health professions education research scientists imbue these aspects of their careers with some of research’s legitimacy. They also are cognizant that their service and teaching activities are important to the institution, even if the institution does not always recognize that value. The participants’ narrative sensemaking maintains their powerful status as researchers but brings service and teaching into the scholarly domain of research—a tactic that can infuse value to non-research activities.

This strategy makes ample sense when considered in light of the status of health professions education in comparison with the disciplines traditionally represented in medical schools and academic teaching hospitals. There are millions of grant dollars available for basic science and medical research. In contrast, there are extremely few grants available for health professions education research and the funding that is available tends to be in the tens-of-thousands and not millions of dollars [23–26]. There are a wide variety of journals that publish research findings from medicine, surgery, nursing, and other clinical specialties, with some having extremely high impact factors (i.e., the New England Journal of Medicine’s impact factor is currently listed as >70) [27]. There are considerably fewer journals that focus on health professions education, and the impact factor of those journals has yet to exceed 5.5 (i.e., it was newsworthy when Academic Medicine’s impact factor achieved 5.255 in 2017) [28]. In many ways, health professions education is a field that has yet to establish itself as an academic discipline [7, 9, 24, 25]. But that credibility will not be secured through multi-million-dollar grants; the money simply is not available. And the development of journals and their impact factors takes years to evolve. Since one currency of academia is research, highlighting the importance of their research efforts and demonstrating how service and teaching activities can contribute to research, the participants contribute to legitimizing the field of health professions education.

However, associating legitimacy with research-oriented measures, and adopting an agenda in accordance with this, is not unique to health professions education scholarship research scientists or health professions education. Due to market-based pressures, many universities (and the departments they house) face pressures to conform to common ideals of accountability and legitimacy [29, 30]. As a result, a faculty member’s career success is often dependent on his/her research productivity, measured by their research topic(s), publications, and grant funding [31–34]. However, scientists working in health professions education are often working across disciplinary boundaries (e.g., a scientist may have a PhD in psychology but is working as a faculty member in a hospital’s department of surgery). Clearly, these researchers are in a unique position—their expertise in research is the skill their organization can harness, not necessarily their disciplinary expertise. To date, research has yet to investigate the sensemaking narratives of clinician educators working in health professions education. It may be that an internal medicine physician working as a faculty member in a university’s or hospital’s department of medicine similarly turns to research for justification and legitimation. The perspectives of clinician educators should be added to the on-going body of research into HPESUs. This is a limitation of the current literature, one that we hope to address in future studies.

Given that sensemaking is contextualized, the generalizability of our findings is limited because they are tied to the Canadian context. However, health professions education scholarship research scientists around the world engage in sensemaking, pulling different arguments together based on the institutional logics that underpin the context and legitimizing the work they do. We contend, therefore, that our research methods and the sensemaking theory can help scholars study the narratives constructed in their local HPESUs, and how legitimacy and power are being leveraged therein. We also suggest that there are institutional logics working in these units that extend beyond the three identified in prior research [1] (e.g., the logic of supporting patient care). We are continuing to develop this program of research to better understand how to support and ensure the longevity of HPESUs and of the field of health professions education.

## Caption Electronic Supplementary Material




