# Supplementary material for: The sensemaking narratives of scientists working in health professions education scholarship units: The Canadian experience
Source: Perspect Med Educ. 2020 May 11;9(3):157–65. doi: 10.1007/s40037-020-00577-1 (PMC7283403; doi:10.1007/s40037-020-00577-1)
Supplement: Supplementary file 1 [file 40037_2020_577_MOESM1_ESM.doc]

**Interview Protocol**

**Sample questions**

| **Question Set #1:**  **Questions about Metrics of Success:** |
| --- |
| 1. Can you describe how you define your success working in Medical Education in your unit? |
| 1.1. To follow up on this question, I’d like to try to explore these criteria of success a little further. Can you describe the criteria of success that are expected of you by your Unit or other external sources? |
| - 1. Can you tell me what supports or enables you to meet these external criteria of success? |
| - 1. Can you describe for me what impedes your ability to meet these external criteria? |
| **Question Set #2:**  **Questions about Culture - Affiliation:** |
| 1. I’d like to ask you about how you conceive of your professional affiliations. Do you feel most affiliated with the Medical Education community or with another community or discipline? |
| **Question Set #3:**  **Questions about Culture - the interactions between Scientists and Clinician Educators:** |
| 1. Next I’d like to discuss with you the people with whom you collaborate. Who are your collaborators? |
| 1. I’d like to talk to you now about clinician educators. By the term “clinician educator”, we mean a very generic description of your clinical colleagues with an appointment in an academic health centre.    1. Think of a few clinician colleagues you work with. Can you tell me in general terms what these collaborations are like?    2. In your mind, does the culture of your institution effect these collaborations? |
| **Question Set #4:**  **Questions about Successful Attainment of the Unit’s Mission** |
| 1. Can you describe the features that you feel are important to enabling the development of a successful unit in Canada? |
| 1. Next I’d like to ask you to think about other features that support the success of units. Given your experiences, what might be some of the contextual or cultural features that a successful unit has? |
